# Regulation of microtubule dynamics by DIAPH3 influences amoeboid tumor cell mechanics and sensitivity to taxanes

**DOI:** 10.1038/srep12136

**Published:** 2015-07-16

**Authors:** Samantha Morley, Sungyong You, Sara Pollan, Jiyoung Choi, Bo Zhou, Martin H. Hager, Kenneth Steadman, Cristiana Spinelli, Kavitha Rajendran, Arkadiusz Gertych, Jayoung Kim, Rosalyn M. Adam, Wei Yang, Ramaswamy Krishnan, Beatrice S. Knudsen, Dolores Di Vizio, Michael R. Freeman

**Affiliations:** 1 Division of Cancer Biology and Therapeutics, Departments of Surgery and Biomedical Sciences, Samuel Oschin Comprehensive Cancer Institute, Cedars-Sinai Medical Center, 8700 Beverly Boulevard, Los Angeles, CA 90048; 2 Urological Diseases Research Center, Boston Children’s Hospital, Boston, MA 02115; 3 Department of Surgery, Harvard Medical School, Boston, MA 02115; 4 Department of Emergency Medicine, Beth Israel Deaconess Medical Center, Boston, MA 02115.

## Abstract

Taxanes are widely employed chemotherapies for patients with metastatic prostate and breast cancer. Here, we show that loss of Diaphanous-related formin-3 (DIAPH3), frequently associated with metastatic breast and prostate cancers, correlates with increased sensitivity to taxanes. DIAPH3 interacted with microtubules (MT), and its loss altered several parameters of MT dynamics as well as decreased polarized force generation, contractility, and response to substrate stiffness. Silencing of DIAPH3 increased the cytotoxic response to taxanes in prostate and breast cancer cell lines. Analysis of drug activity for tubulin-targeted agents in the NCI-60 cell line panel revealed a uniform positive correlation between reduced DIAPH3 expression and drug sensitivity. Low DIAPH3 expression correlated with improved relapse-free survival in breast cancer patients treated with chemotherapeutic regimens containing taxanes. Our results suggest that inhibition of MT stability arising from DIAPH3 downregulation enhances susceptibility to MT poisons, and that the DIAPH3 network potentially reports taxane sensitivity in human tumors.

Metastatic dissemination is a multistep process that involves cell migration, invasion and growth at distant sites. The ‘amoeboid’ phenotype has emerged as a migratory mechanism that facilitates metastasis^1^,^2^. Amoeboid behavior is prominent at the invasive front of tumors^3^,^4^, confers rapid migration rates^1^,^2^,^5^,^6^, and enables survival within the vasculature^7^. Collectively, these malignant features suggest that amoeboid cells are highly aggressive tumor cell variants that potentially evolve subsequent to an epithelial-to-mesenchymal transition (EMT^8^).

Tumor cells with amoeboid features display limited dependence on proteolysis and navigate through tissue spaces by rapidly deforming their shape^1^,^2^. Other characteristics of amoeboid cells include enhanced actomyosin contractility mediated by Rho kinase (ROCK) signaling, diminished adhesion, heightened chemotactic responses, and dynamic membrane blebbing^9^,^10^,^11^,^12^. Amoeboid morphology is regulated by growth factor-, cytokine-, and MMP-dependent signaling, transcriptional reprogramming, and cytoskeletal alterations^1^,^2^,^9^,^13^,^14^,^15^,^16^,^17^. Methods of identifying amoeboid cells *in vivo*, and an understanding of their vulnerabilities to chemotherapies, could provide novel opportunities for therapeutic intervention.

We identified the Diaphanous-related formin-3 (DIAPH3), a member of the formin family of cytoskeletal regulators^18^, as a key mediator of amoeboid behavior^5^,^19^. We reported that DIAPH3 silencing alters the microtubule (MT) cytoskeleton, impairs endocytic trafficking, augments signaling through the EGFR/MEK/ERK axis, and induces resistance to the EGFR inhibitor gefitinib. Signaling pathway activation culminates in enhanced motility, invasion, and experimental metastasis in mice. DIAPH3 silencing also promotes the shedding of extracellular vesicles capable of altering the tumor microenvironment^6^,^19^,^20^.

MT exist as either dynamic or stable populations, distinguished by their turnover rate^21^,^22^. Slowed depolymerization of dynamic MT, through interaction with MT-associated proteins, such as formins, results in MT stabilization. The longevity of these stable MT populations results in accumulation of diverse post-translational modifications, including acetylation^21^,^22^. Mounting evidence correlates reduced MT stability with a mesenchymal-amoeboid transition (MAT)^5^,^14^,^15^,^16^,^17^, suggesting that alteration in the MT lifecycle significantly contributes to the onset of this phenotype.

Agents that disrupt MT dynamics are among the most frequently employed chemotherapies. Paclitaxel, docetaxel and epothilone B are MT-stabilizing agents (MSA) that prevent MT disassembly and induce apoptosis through prolonged cell cycle arrest at G2/M^23^. While impairment of spindle dynamics is the best-characterized mode of action of MSA^24^, these agents also impinge on interphase MT^25^ or elicit tumor cell death through MT-independent pathways^26^. These compounds are commonly used to treat patients with advanced metastatic prostate or breast cancers (PCa, BCa)^27^,^28^.

Neo-adjuvant chemotherapy has been demonstrated to improve post-operative outcomes in cancer patients. However, response to treatment is variable^29^,^30^, underscoring the need for molecular predictors of chemo-sensitivity^31^,^32^. The DLDA-30 signature is a pharmacogenomic predictor developed to identify BCa patients who are likely to achieve a pathologic complete response (pCR) to a pre-operative regimen of taxanes, fluorouracil, anthracyclines, and cyclophosphamide (T/FAC therapy)^33^. The signature displays greater predictive power to detect pCR in patients treated with taxanes plus FAC than FAC alone^34^. The activity of neoadjuvant taxanes in BCa has also been tested in the I-SPY 1 trial, in which chemo-responsiveness was predicted through imaging, clinical, and genomic measurements. Combining genomic signature scores with receptor status improved prediction of pCR^29^ and recurrence-free survival (RFS)^30^. In these and other trials^35^, the response to neo-adjuvant treatment predicted long-term outcomes, suggesting that refinements of biomarker signatures would improve the selection of chemotherapy to treat recurrent disease.

The present study was undertaken to assess the functional ramifications of the cytoskeletal defects associated with DIAPH3 loss, and to determine their potential clinical significance. Here we show that DIAPH3 silencing alters MT stability and dynamics, and increases sensitivity to MSA. These findings identify a novel chemotherapeutic vulnerability in tumor cells with amoeboid features arising from disruption of the cytoskeleton.

## Results

### DIAPH3 loss correlates with shorter overall survival

We previously reported an increased frequency of *DIAPH3* loss in patients with metastatic disease^5^,^19^. DIAPH3 silencing in preclinical models promoted amoeboid features, migration and invasion, and experimental metastasis^5^,^19^. Conversely, enforced expression suppressed amoeboid characteristics and promoted mesenchymal features, including upregulation of N-cadherin, actin stress fibers, and lamellipodia, suggesting that DIAPH3 is a node capable of regulating the transition between amoeboid and mesenchymal phenotypes.

**Figure 1:**
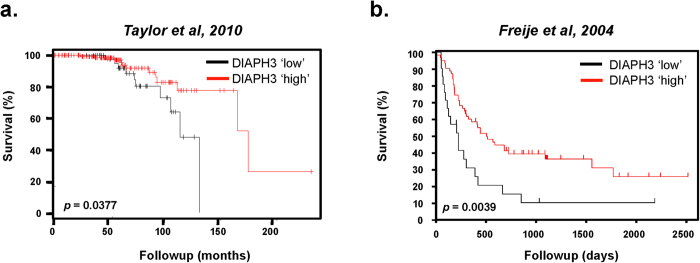
Low DIAPH3 expression is associated with reduced patient survival . **A**. Kaplan-Meier analysis of overall survival in PCa patients^36^ whose tumors are ‘DIAPH3 low’ (<25^th^ percentile, mRNA expression) versus ‘DIAPH3 high’ (≥25^th^ percentile, mRNA expression). Log rank test, p = 0.0377. **B**. Kaplan-Meier analysis of overall survival in glioblastoma patients whose tumors display ‘DIAPH3 low’ versus ‘DIAPH3 high’ expression^37^. Log rank test, p = 0.0039.

Consistent with this notion, DIAPH3 loss suppressed EMT-like features. DIAPH3 silencing attenuated expression of N-cadherin (Supplementary Fig. S1A), E-cadherin (Supplementary Fig. S1B–C), and β-catenin (Supplementary Fig. S1B,D). Loss of E-cadherin is observed during the ‘cadherin switch’ of EMT^8^. However, loss of this epithelial marker is classically accompanied by upregulation of the ectopic mesenchymal marker N-cadherin^8^. That expression of both N-cadherin and E-cadherin are reduced by DIAPH3 loss implies that transition to an amoeboid phenotype can occur after cells have progressed through an EMT. Consistent with previous reports^5^,^19^, these findings suggest that DIAPH3 silencing promotes features of heightened tumor cell aggressiveness. Analysis of DIAPH3 expression in human cohorts supports this hypothesis. PCa patients^36^ with ‘low’ intratumoral DIAPH3 exhibited significantly shorter OS times as compared to those with ‘high’ expression (Fig. 1A). Similarly, worsened survival rates were detected in a cohort of glioblastoma patients (Fig. 1B,^37^) with ‘low’ DIAPH3 expression. These observations suggest DIAPH3 loss may be of clinical significance and relevant to patient prognosis.

### DIAPH3 loss decreases MT stability and alters global MT topology

**Figure 2:**
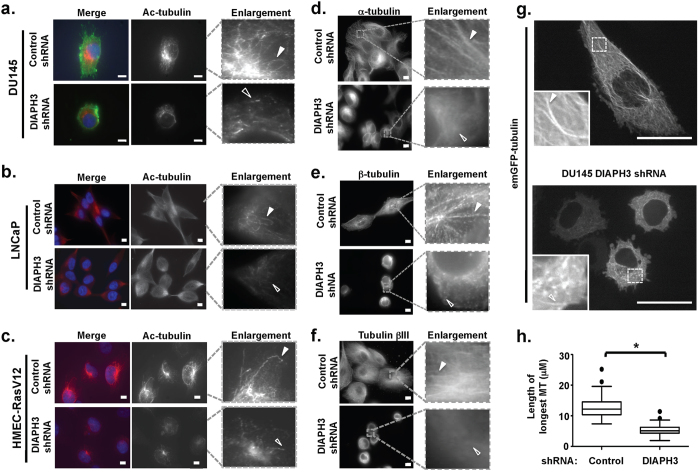
DIAPH3 silencing reduces the population of stable MT and alters MT topology. **A.** DIAPH3-silenced and control DU145 cells, plated on collagen I, were stained with Cholera toxin B and Ac-tubulin. Filled arrowheads indicate long MT polymers, open arrowheads indicate short MT polymers/fragments. Scale bar, 10 μm. **B**. DIAPH3-silenced LNCaP cells or control cells, stained with Ac-tubulin antibodies. Scale bar, 10 μm. **C.** DIAPH3-silenced HMEC-RasV12 cells or control cells, stained with Ac-tubulin antibodies. Scale bar, 10 μm. **D-F**. DIAPH3-silenced DU145 cells or controls, plated on glass coverslips, were stained with antibodies against α-tubulin **(D)**, β-tubulin **(E)**, or tubulin βIII **(F)**. Data shown are representative of at least 10 fields per condition, in two independent experiments. **G.** DU145 cells (control, *top*, or DIAPH3-silenced, *bottom*) were infected with 100 μl of CellLight GFP-tubulin, incubated for 1-2 days and imaged by spinning disc confocal microscopy. Scale bar, 25 μm. **H**. The median length of the longest MT in control vs. DIAPH3-silenced DU145 cells. The freehand line function of ImageJ was used to trace the length of the 2 to 3 longest MT in a single cell expressing emGFP-tubulin. A total of 41 MT in control and 37 MT in DIAPH3-silenced cells were measured. Length was converted from pixels to microns, and median length values for each cell type determined. Similar results were obtained from cells expressing TagRFP-tubulin. Mann Whitney test, p < 0.001. Filled arrowheads indicate long MT polymers, open arrowheads indicate short MT polymers/fragments.

DIAPH3 loss can perturb the MT cytoskeleton^5^. To better understand the significance of this association, we assessed the effect of DIAPH3 loss on MT stability, using Ac-tubulin as a marker of stable MT^21^,^22^. DIAPH3 deficiency induced shortened acetylated MT in DU145 and LNCaP PCa cells, and in HRAS-transformed HMEC cells. This effect was accompanied by reduced Ac-tubulin levels (Fig. 2A–C, Supplementary Fig. S2A–D,^5^). Stable MT reformation following cold-induced depolymerization^38^ was also attenuated by DIAPH3 silencing (data not shown). Concordantly, enforced expression of GFP-DIAPH3 increased Ac-tubulin levels (Supplementary Fig. S2E,^5^). These findings suggest that DIAPH3 loss alters MT architecture and reduces MT stability, thereby implicating DIAPH3 as a MT-stabilizing protein.

Because only a subpopulation of MT are acetylated/stable^21^,^22^, we also examined the total MT pool. Similarly to observations for Ac-tubulin, MT polymers containing α-tubulin (Fig. 2D), β-tubulin (Fig. 2E), or the isotype tubulin βIII (Fig. 2F), were reduced in length in DIAPH3-deficient cells compared to control cells. Quantitation of the longest MT lengths in cells with enforced expression of GFP-tubulin (Fig. 2G–H) confirmed this reduction. No differences in expression levels of unmodified tubulins were detected by immunoblotting^5^. Taken together, these findings implicate DIAPH3 in regulation of the MT life cycle.

### DIAPH3 loss increases MT dynamics

**Figure 3:**
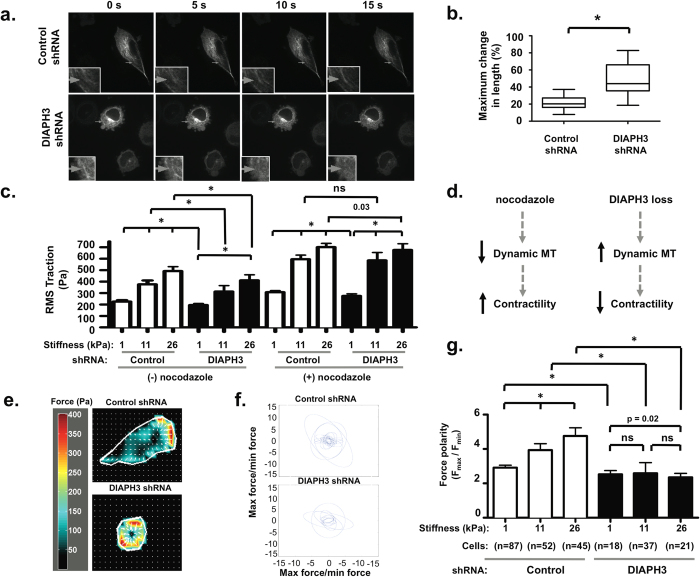
The population of dynamic MT is increased by DIAPH3 loss. **A.** Montage of TagRFP-tubulin visualized by spinning disc confocal microscopy. Note relative persistence of example MT in control cell (inset, top), yet rapid disappearance of example MT in DIAPH3-silenced cell (inset, bottom), indicating increased MT dynamics. **B.** Quantitation of the maximum MT length change in DU145 cells. MT length was measured over 10 frames (30 s period), and the maximal length change calculated as described in the Methods. The median of the maximum MT length change in control or DIAPH3-silenced cells is shown as a Tukey plot, and was analyzed with a Mann-Whitney test. **C**. TFM measurements of RMS value of traction in control or DIAPH3-silenced cells, with and without incubation with 2 μM nocodazole. Note increased contractility in response to nocodazole in both cell lines, but to a greater extent in DIAPH3-deficient cells, especially at 11 and 26 kPa stiff substrates, at which tractions were of comparable magnitude between control and DIAPH3-silenced cells. * = p < 0.0001, Student’s t-test. **D.** Schematic summarizing effects of DIAPH3 silencing or nocodazole on contractile force. Dynamic MT inhibit contractility^40^,^41^. MT depolymerization with nocodazole disrupts MT, and increases traction (left). DIAPH3 loss, by increasing dynamic MT, is predicted to decrease contractility (right). **E**. Representative contraction maps, demonstrating the location and magnitude of traction exerted by control and DIAPH3-silenced DU145 cells. Note asymmetry of forces in control cells versus lack of force polarity in DIAPH3-deficient cells. **F**. Representation of traction orientation and polarity. For each ellipse, representing a single cell, semi-axes are determined by the eigenvalues of the matrix comprised by the first-order moment of the traction (M) and orientation determined by the corresponding eigenvectors of M. Note the greater circular contour (symmetry) of DIAPH3-deficient cells, indicating reduced traction polarity relative to more elliptical (polarized asymmetry of) control cells. **G.** Traction polarity, obtained from the ratio of the eigenvalues of M in control or DIAPH3-silenced DU145 cells at the indicated substrate stiffnesses. Note increased traction polarity with increasing substrate stiffness in control cells, yet relative insensitivity to substrate stiffness in DIAPH3-deficient cells.

We next employed live cell imaging of fluorescent tubulin to assess whether reduced MT stability was accompanied by altered MT dynamics. MT length in control DU145 cells was relatively constant over a period of 15 s (Fig. 3A), whereas it changed rapidly in DIAPH3-deficient cells, with faster disappearance (inset), or more rapid elongation, during the same timeframe (Fig. 3A). The maximum change in MT length, quantified over a period of 30 s, was more pronounced in DIAPH3-silenced cells (Fig. 3B). This greater change in MT length was observed when monitoring both emGFP-tubulin (Fig. 3B, Supplementary Movies 1–4) and TagRFP-tubulin. Together, these findings suggest that loss of DIAPH3 increases the fraction of dynamic MT. To our knowledge, this is the first demonstration of increased MT dynamics in cells displaying amoeboid features.

To further characterize amoeboid mechanics, we utilized traction force microscopy (TFM), which monitors the contractile force exerted by a cell on an elastic substrate^39^. Cell traction increased with substrate stiffness in both control and DIAPH3-deficient cells (Fig. 3C). However, these traction forces were reduced, modestly albeit significantly, in DIAPH3-silenced cells (Fig. 3C). DIAPH3 loss also greatly reduced overall contractile strength (Supplementary Fig. S3A), as detected when computing the cell contractile moment (Supplementary Fig. S3A), an integrated measure of traction and cell spreading area (Supplementary Fig. S3B). Following treatment with the MT depolymerizing agent nocodazole, traction forces were enhanced in both control and DIAPH3-silenced cells but to a greater extent in the latter, especially at 11 and 26 kPa stiff substrates. Importantly, tractions were now comparable between control and DIAPH3-silenced cells. Because dynamic MT counterbalance cell contractility^40^,^41^ (Fig. 3D), this lack of difference in the nocodazole condition supports the contribution of dynamic MT in mediating the mechanical effects of DIAPH3 loss.

Next, we characterized traction orientation and polarity. We observed that the traction forces exerted by control cells were polarized and increasingly asymmetric with substrate stiffness (Fig. 3E–G), while they were evenly oriented in cells lacking DIAPH3 (Fig. 3E–F). In the latter, traction polarity was also invariant across substrate stiffness (Fig. 3G). These results suggest that DIAPH3 silencing influences both the extent and polarity of contractile force, as well as the ability of cells to polarize in response to matrix rigidity. These data reveal a novel role for formin proteins in regulating mechanical responsiveness to substrate stiffening.

### DIAPH3 interacts with MT

**Figure 4:**
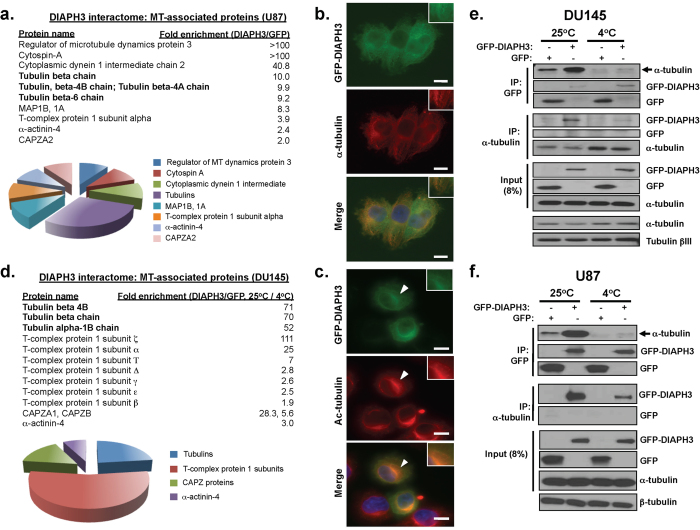
DIAPH3 interacts with MT. **A.** MT-relevant interactome of DIAPH3 in U87 cells. Cells stably expressing GFP- or GFP-DIAPH3 were immunoprecipitated with antibodies against GFP, and subjected to proteomic analysis by tandem LC-MS/MS. The fold-enrichment of interaction with GFP-DIAPH3 over GFP in each protein is shown. Note prevalence of tubulins as DIAPH3-interacting proteins. The pie chart illustrates the fraction of each subtype of MT-relevant proteins to the total number of MT-relevant proteins detected. **B.** Co-localization of DIAPH3 and α-tubulin in DU145 cells stably expressing GFP-DIAPH3. Scale bar, 10 μm. **C**. Co-localization of DIAPH3 and Ac-tubulin in DU145 cells stably expressing GFP-DIAPH3. Scale bar, 10 μm. **D.** Enrichment of DIAPH3 interaction with MT-relevant proteins, when MT are intact (25 ^o^C) vs. depolymerized (4 ^o^C), in DU145 cells. After immunoprecipitation from cells stably expressing GFP- or GFP-DIAPH3, interacting proteins were analyzed by LC-MS/MS. The fold-enrichment at which each protein interacted with GFP-DIAPH3 or GFP at 25 ^o^C vs. 4 ^o^C was determined. *Bottom,* pie chart illustrating the fraction of each subtype of MT-relevant proteins to the total number of MT-relevant proteins detected. **E-F.** Interaction of DIAPH3 with α-tubulin. Reciprocal co-immunoprecipitations of GFP-DIAPH3 or GFP with α-tubulin in DU145 (E) or U87 (F) cells was performed at 25 ^o^C or 4 ^o^C. Note greater extent of binding of GFP-DIAPH3 to α-tubulin, at 25 ^o^C vs. 4 ^o^C, despite equivalent precipitation of bait at these two temperatures.

Given the findings above implicating DIAPH3 in regulation of MT dynamics, and of the reciprocity between DIAPH3 and MT in regulating cell contractility, we sought to further understand the DIAPH3-MT relationship. We performed an unbiased proteomic analysis to identify DIAPH3-interacting proteins. Initially we used U87 cells, a glioblastoma cell line displaying amoeboid properties (Supplementary Fig. S4) that can be attenuated by enforced expression of DIAPH3^5^. Lysates from U87 cells stably expressing GFP or GFP-DIAPH3 were immunoprecipitated with antibodies to GFP and subjected to liquid chromatography/tandem mass spectrometry (LC-MS/MS). Of 417 proteins identified, 130 displayed 2-fold greater binding to GFP-DIAPH3 as compared to GFP alone. Multiple tubulin isotypes and MT-associated proteins preferentially precipitated with DIAPH3 in U87 cells (Fig. 4A). Consistent with this, DIAPH3 bound to α-tubulin during reciprocal co-immunoprecipitation (Supplementary Fig. S5A), and co-localized with α-tubulin (Fig. 4B) in DU145 cells. DIAPH3 also co-localized (Fig. 4C) and co-immunoprecipitated (Supplementary Fig. S5B) with Ac-tubulin. Thus, DIAPH3 appears to associate with MT, including a subpopulation of stable (acetylated) MT.

Because these findings suggest that DIAPH3 promotes MT stability, a process occurring at later stages of the MT lifecycle (after MT polymerization), we next asked whether DIAPH3 preferentially forms complexes with polymerized rather than soluble tubulin. To do this, we assessed the effect of temperature on the DIAPH3 interactome. At 25 ^o^C MT polymers remain intact, while at 4 ^o^C the MT lattice depolymerizes into soluble tubulin heterodimers^38^. Thus, lysates from DU145 cells, stably expressing GFP or GFP-DIAPH3, were immunoprecipitated at 25 ^o^C or 4 ^o^C to preserve or disrupt MT architecture, respectively. LC-MS/MS confirmed interaction of DIAPH3 with tubulins, which was enhanced at 25 ^o^C compared to 4 ^o^C (Fig. 4D, Supplementary Fig. S5C–D). The proteins identified by mass spectrometry as preferentially precipitating with DIAPH3 versus GFP (differentially-expressed proteins, DEPs) at 25 ^o^C were significantly associated with three Gene Ontology (GO) processes (Supplementary Fig. S5E), including ‘MT-based processes’ (~20% of total proteins). Similarly, DEPs involved in ‘cytoskeletal organization’ associating with DIAPH3 were higher at 25 ^o^C (15%) than 4 ^o^C (~7%, Supplementary Fig. S5E–F). These findings support a greater interaction of DIAPH3 with the MT cytoskeleton over soluble tubulin heterodimers. This interpretation was confirmed by reciprocal co-immunoprecipitation of DIAPH3 and α-tubulin in DU145 (Fig. 4E) and U87 (Fig. 4F) cells. In both cell types, DIAPH3 also interacted with MT-regulatory and stabilizing proteins (Fig. 4A,D, and Supplementary Fig. 5C–D), some of which were common between the two cell lines. These findings support a preferential association of DIAPH3 with polymerized tubulins.

### DIAPH3 silencing increases responsiveness to taxanes and epothilone B

**Figure 5:**
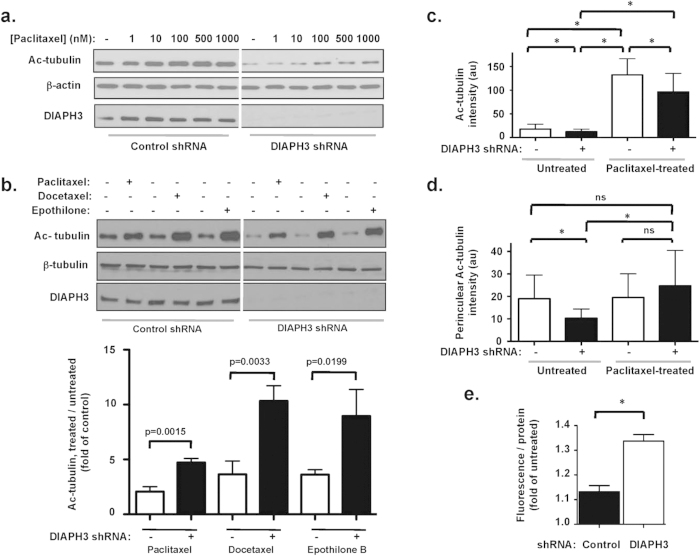
DIAPH3 silencing increases MT responsiveness to MT stabilizing agents. **A.** DU145 cells were treated with varying concentrations of paclitaxel for 30 min prior to lysis and immunoblotting with Ac-tubulin antibodies. **B**. *Top,* cells were treated with 500 nM of each MSA for 30 min at 37 ^o^C, and assessed for Ac-tubulin levels. *Bottom*, data were quantified after normalization of Ac-tubulin to β-tubulin intensities, followed by ratiometric comparison between MSA-treated and untreated conditions. Note greater fold-change in Ac-tubulin levels in DIAPH3-deficient cells following treatment with MSA relative to baseline (untreated) conditions. Data shown are average ± SD from 3 combined, independent trials. **C**. Quantitation of Ac-tubulin fluorescence, from at least 25 cells per condition. Note that the reduced Ac-tubulin fluorescence in cells silenced for DIAPH3, relative to controls, persists in the presence of taxol. **D**. Quantitation of perinuclear Ac-tubulin fluorescence, from at least 25 cells per condition. Note the greater fold-increase in fluorescence by MSA treatment (relative to untreated conditions) in DIAPH3-deficient cells. A (*) indicates p < 0.0001. **E**. Intracellular accumulation of OG-PTX in cells expressing or silenced for DIAPH3 was monitored spectrophotometrically, and fluorescence normalized to protein concentration in each well. n = 2 independent trials.

Next we examined how DIAPH3 silencing affects MT responses to MSA. Immunoblotting showed that in the absence of MSA, Ac-tubulin levels were reduced by DIAPH3 silencing (Fig. 5A), consistent with data in Fig. 2. Paclitaxel increased tubulin acetylation in a dose-dependent fashion in both control and DIAPH3-silenced cells. Similar dose-dependent inductions were observed by docetaxel or epothilone B (data not shown). With DIAPH3 knockdown, Ac-tubulin levels at all MSA concentrations remained below those of controls, consistent with higher levels of dynamic MT. Furthermore, the fold-change in Ac-tubulin levels in response to MSA treatment was substantially greater when DIAPH3 was silenced (Fig. 5B). This greater magnitude of change in MT stability is consistent with a greater extent of dynamic MT in DIAPH3-silenced cells. It is also suggestive that cells lacking DIAPH3 are more sensitive to MSA.

Quantifying intracellular Ac-tubulin intensities, using immunofluorescence and 3D computational modeling^42^, yielded analogous results. DIAPH3 silencing reduced basal Ac-tubulin levels relative to those of control cells (Fig. 5C). This reduction persisted during paclitaxel treatment (Fig. 5C). In interphase cells, acetylated MT are predominantly perinuclear^43^ (e.g. Fig. 4C). DIAPH3 silencing reduced Ac-tubulin intensity in the perinuclear region (Fig. 5D). Paclitaxel did not appreciably alter this intensity in control cells, but increased perinuclear Ac-tubulin levels in DIAPH3-deficient cells (Fig. 5D). These findings suggest a greater degree of stabilization of MT by MSA when DIAPH3 is silenced.

**Figure 6:**
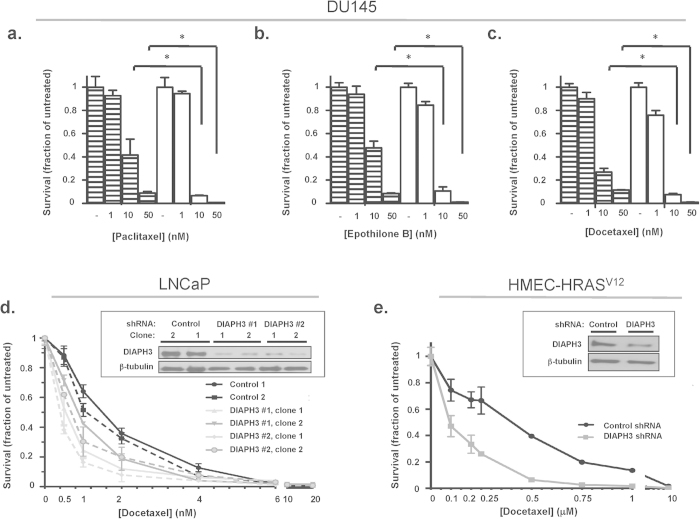
Greater cytotoxicity of MT-stabilizing agents in cells silenced for DIAPH3. **A-C.** DU145 cells expressing or silenced for DIAPH3 were incubated with varying concentrations of each MSA for 4 d, and cell survival monitored as crystal violet absorbance. Values are normalized to vehicle (DMSO) treatment. **A** (*) indicates a p-value of <0.02 (Student’s t-test). **D.** LNCaP cells expressing or stably silenced for DIAPH3 were treated with the indicated concentrations of docetaxel for 4 d, and survival assessed as in (**A-C**). Inset, silencing of DIAPH3. **E.** HMEC-RasV12 cells expressing or stably silenced for DIAPH3 were treated with the indicated concentrations of docetaxel and assessed as in (**D**). Inset, silencing of DIAPH3. n = 2 independent trials.

DIAPH3 depletion also increased intracellular retention of fluorescent Oregon Green 488-paclitaxel (Fig. 5E), suggesting that DIAPH3 loss increases the intracellular concentration of MSA. Consistent with greater activity, survival of DU145 cells in the presence of paclitaxel, docetaxel or epothilone B was significantly reduced by DIAPH3 loss, as evidenced by reduced cell number (Fig. 6A–C). DIAPH3 loss also sensitized androgen-dependent LNCaP PCa cells to taxanes (Fig. 6D), suggesting mechanisms at least partially independent of androgen receptor activity^26^,^44^,^45^,^46^. Similar results were seen using HMEC-RasV12 breast cancer cells in which DIAPH3 was silenced (Fig. 6E).

### Low DIAPH3 expression is associated with greater sensitivity to MT-directed chemotherapy drugs

To assess the potential generality of the association between low levels of DIAPH3 and sensitivity to taxanes and other MT-directed drugs, we performed COMPARE analysis using the CellMiner™ tool^47^. We calculated pair-wise correlations between cytotoxicity Z-scores derived from GI_50_ values (the concentration that causes 50% Growth Inhibition) and gene expression Z-Score patterns within the NCI-60 cell line panel. Using NCI Anticancer Drug Screen’s Standard Agents Database^47^, we selected agents known to target tubulin, which were then classified as either stabilizing or depolymerizing. Compounds unable to be categorized into these groups were excluded from analysis. As shown in Supplementary Table S1, cells with low DIAPH3 expression uniformly exhibited increased sensitivity to these agents (Pearson’s correlation less than −0.25 in 7 agents). The closely-related genes DIAPH1 and DIAPH2 did not show this relationship; DIAPH1 in fact exhibited an inverse correlation (increased resistance with lower gene expression; Pearson’s correlation ≥0.25 for 8 MT-targeting agents). DIAPH2 did not show any recognizable sensitivity pattern. When performing the same analysis against 75 known Topoisomerase I-targeting agents, no significant correlations or sensitivity patterns were detected. Taken together, these data suggest that sensitivity to MT-targeted agents from reduced gene expression is unique to DIAPH3 within the Diaphanous formin family.

### Low DIAPH3 expression correlates with response to taxane-containing chemotherapy in BCa patients

Given the above findings of *in vitro* chemosensitivity, we assessed whether DIAPH3 loss was associated with MT-directed chemotherapeutic response in patients. We interrogated gene expression profiles from three randomized clinical trials in BCa patients designed to test taxane-containing regimens in the neoadjuvant setting^29^,^33^,^34^,^35^. Microarray data were available from 111 BCa patients in trial 1^29^, 91 patients in trial 2^34^, and 310 patients in trial 3^35^, from which outcomes measures including pCR, RFS and OS following taxane-containing chemotherapy were assessed (Supplementary Table S2).

**Figure 7:**
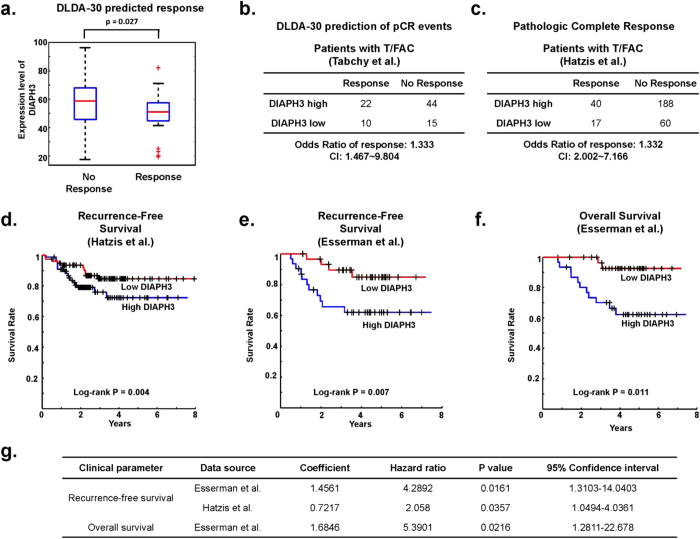
Low DIAPH3 expression is associated with improved clinical outcomes in breast cancer patients after taxane-containing chemotherapy. **A** and **B**. DIAPH3 levels differ in responders and non-responders to neo-adjuvant T/FAC chemotherapy, as predicted by expression of the DLDA-30 signature^34^. **A**. The median expression level of DIAPH3 is shown as a box and whisker plot. **B**. Fisher’s exact test demonstrates increased chemotherapeutic response in patients with low median DIAPH3 expression^34^. **C**. DIAPH3 levels are lower in cancers with a complete pathologic response (pCR) based on histopathological inspection. Contingency table demonstrating the frequency of clinically-achieved pCRs in patients treated with T/FAC therapy. Cox proportional hazards analysis revealed a significant association of low DIAPH3 expression with therapeutic responsiveness^35^. **D-F**. Kaplan-Meier curves demonstrating that low DIAPH3 expression is associated with a longer time to recurrence (**D**,^35^ and **E**,^29^) and with greater overall survival (**F**,^29^) in patients treated with taxane-containing neoadjuvant chemotherapy. **G**. Cox proportional hazards regression analyses, derived from datasets used in panels **D-F**, demonstrating increased hazards ratios (improved response) for recurrence-free and overall survival status in patients with low DIAPH3 expression.

The DLDA-30 signature^33^,^34^ predicted the occurrence of a pCR in patients with low intratumoral DIAPH3 levels (Fig. 7A). Similarly, pCR was predicted to occur with greater frequency in ‘DIAPH3 low’ cancers (Fig. 7B). Consistent with this prediction, histologically-confirmed pCR was achieved with greater frequency following neoadjuvant chemotherapy (Fig. 7C,^35^). Because pCR correlates with RFS^30^, we examined whether DIAPH3 expression correlated with time to progression. Univariate analyses^35^ revealed that low levels of DIAPH3 mRNA correlated with extended RFS in cohorts from 2 independent clinical trials (Fig. 7D,E,G). Similarly, in the I-SPY 1 trial^29^, prolonged OS (Fig. 7F,G) was observed in patients with ‘DIAPH3 low’ cancers treated with T/FAC chemotherapy.

**Figure 8:**
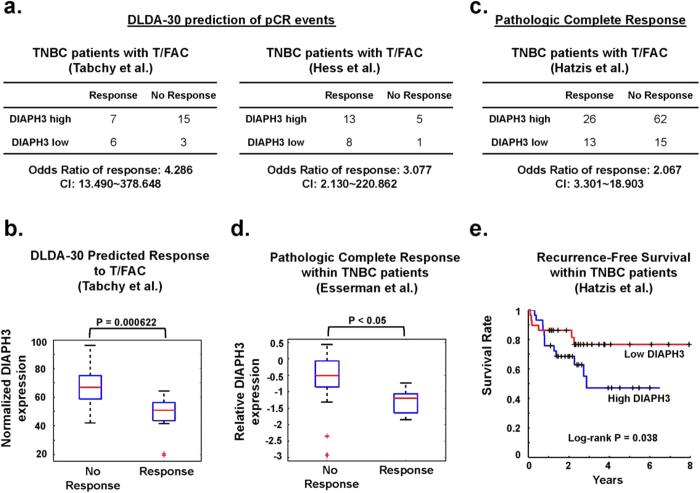
Greater pathologic complete response (pCR) after treatment with neoadjuvant taxane-containing therapy in TNBC expressing low DIAPH3. **A**. Prediction of a pCR in 2 cohorts of TNBC patients whose cancers display low DIAPH3 expression^33^,^34^. **B**. DIAPH3 expression is lower in TNBC patients predicted to respond to T/FAC therapy than those predicted not to respond^34^. **C**. Achievement of a histologically-defined pCR in cancers with low DIAPH3 levels^35^. Cox proportional hazards regression analysis revealed significant association between low DIAPH3 expression and pCR. **D**. Lower median DIAPH3 expression in patients achieving a pCR^29^. **E**. Kaplan-Meier analyses demonstrating a trend toward extended time to recurrence in patients with low DIAPH3 expression^35^.

Next we determined whether the negative association between DIAPH3 and clinical outcome following neoadjuvant chemotherapy was greater in triple-negative BCa (TNBC) patients. In two TNBC cohorts^33^,^34^, pCR predicted by the DLDA-30 signature was greater when DIAPH3 expression was low (Fig. 8A). Similarly, in patients predicted by the DLDA-30 signature to respond to neoadjuvant T/FAC chemotherapy, relative to those predicted not to do so, median DIAPH3 expression was significantly lower (Fig. 8B). When using histologically-confirmed pCR as the outcome, patients with low intratumoral DIAPH3 expression exhibited a greater frequency of response (Fig. 8C,D) following chemotherapy and longer RFS (Fig. 8E). These findings suggest that patients with low intratumor DIAPH3 expression are more responsive to taxane-based chemotherapy regimens.

## Discussion

This study has identified a novel link between molecular mechanisms underlying amoeboid behavior, cytoskeletal dynamics and sensitivity to MT-directed chemotherapy. We previously reported that DIAPH3 depletion promotes invasive, amoeboid features in multiple tumor cell backgrounds^5^. Here we present evidence that amoeboid behaviors elicited by DIAPH3 loss likely occur as a result of disruption of the MT cytoskeleton. This conclusion is supported by assessments of MT stability and dynamics; quantitative measurements of polarized force generation, contractility and response to substrate stiffness; and identification of likely members of DIAPH3 protein complexes by LC-MS/MS. DIAPH3 down-regulation also sensitized cancer cells to growth suppression by MT-targeting chemotherapies. To our knowledge, this represents the first therapeutic vulnerability of amoeboid cells to agents routinely used in clinical practice for aggressive disease.

Some tumor cells can interconvert between mesenchymal and amoeboid modes in order to navigate changing environmental contexts. This plasticity provides challenges for therapeutic strategies targeting metastatic tumors. Our study demonstrates that cells with EMT features contain a greater number of stable MT (Figs 2,5), which are less responsive to taxane-induced stabilization, than cell displaying amoeboid characteristics. These cells were also less sensitive to taxane-induced cytotoxicity than amoeboid cells (Fig. 6). Thus, DIAPH3 loss appears not only to induce MAT but also to enhance MT-responsiveness and susceptibility to taxanes, leading to greater drug-induced cell death. These findings are of interest in light of reports that EMT confers taxane resistance^48^. These observations highlight the importance of considering mechanisms of tumor cell plasticity when evaluating chemotherapeutic efficacy in the context of precision medicine.

The androgen receptor (AR) is a transcription factor that plays a key role in PCa pathogenesis, in part through induction of EMT^45^. Several reports provide compelling evidence that MT dynamics control AR intracellular trafficking^44^,^45^,^46^. Taxane-induced MT stabilization suppresses AR nuclear translocation, precluding AR promoter occupancy and transcriptional activity, and evoking cell death. In patients with CRPC, reduced AR nuclear localization correlates with patient response to taxane-based chemotherapy^44^. These reports highlight an importance of MT dynamics for AR-dependent prostate tumorigenesis, and may explain the observed clinical benefit of neoadjuvant taxanes prior to radical prostatectomy^49^. These reports also underscore a role for MT stability in taxane responsiveness in cells having undergone EMT; our findings highlight that MT additionally contribute to the responsiveness of amoeboid cells to these agents. We report that both androgen-dependent LNCaP cells and castrate-resistant DU145 cells are sensitized to taxane-induced cytotoxicity by DIAPH3 loss (Fig. 6). Thus, DIAPH3 loss augments the anti-neoplastic effects of taxanes through pleiotropic, and in some cases androgen-independent, mechanisms. These data raise the intriguing possibility that taxanes could be of utility in AR-independent prostate tumors with low DIAPH3.

DIAPH3 appears to act as a MT-stabilizing protein, suppressing MT turnover. While the role of the actin cytoskeleton in determining the mechanical properties of cells is widely appreciated, the contribution of MT has been much less studied, especially in amoeboid cells. Using TFM, we observed that DIAPH3 loss reduced contractile forces generated by DU145 cells, and this reduction of traction was reversed by nocodazole, suggesting that dynamic MT contribute substantially to the magnitude of force generation. This was observed across a range of physiologically relevant stiffnesses, suggesting that reduced traction by amoeboid tumor cells can facilitate movement through diverse tissue spaces. These findings are consistent with reports that fast-migrating keratocytes and neutrophils display lower tractions than those of highly adherent cells^50^,^51^.

**Figure 9:**
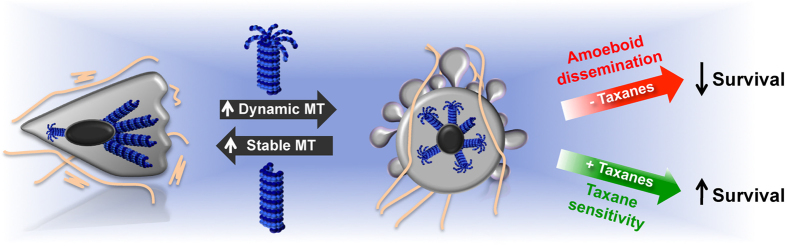
Model for the association of DIAPH3 loss with amoeboid motility and taxane sensitivity. Low DIAPH3 expression reduces the extent of MT stability, and in turn increases dynamic MT content. These transitions may lead to either 1) amoeboid behavior and worse prognosis (in untreated patients) or 2) taxane sensitivity and better prognosis (in taxane-treated patients).

In summary, the results of this study show that DIAPH3 loss can mediate a transition to a more aggressive phenotype, which can arise from cells that have undergone EMT. Ironically, DIAPH3 loss also sensitizes cells to MT-directed chemotherapy. These conclusions are diagramed in Fig. 9. DIAPH3 stabilizes MT, while DIAPH3 downregulation increases MT turnover and dynamics, leading to reduced cell contractility and a more uniform distribution of traction forces. This phenotypic transition makes the tumor cells more sensitive to taxanes and other MT-targeted agents. Johansson *et al.* suggest DIAPH3 loss to be a candidate driver of BCa^52^. Consistent with this, we find that DIAPH3 loss correlates with worse prognosis in multiple cancer cohorts. The association of low DIAPH3 expression with both metastatic disease and shorter overall survival suggests that amoeboid behavior promotes metastatic dissemination. Our present findings suggest that profiling of tumors for DIAPH3 loss and related network perturbations, including levels of Ac-tubulin, may have predictive value in selecting rational treatment strategies for aggressive disease.

## Material and Methods

### Cell lines and reagents

Parental and DIAPH3-silenced or over-expressing cell lines, such as DU145, Human Mammary Epithelial (HMEC), or U87, have been described^5^. Paclitaxel (Cayman Chemicals, Sigma), epothilone B (Cayman Chemicals), and docetaxel (LC Laboratories). Cholera toxin B (CTxB, Sigma, Life Technologies). β-octylglucopyranoside and cacodylate buffer (Sigma), Protein A/G Agarose (Santa Cruz Biotechnology), and Oregon Green-488 Paclitaxel (Invitrogen). Antibodies: Ac-tubulin (Abcam, Cell Signaling), GFP (Genscript), Cleaved PARP, E-cadherin, β-catenin, (Cell Signaling), α-tubulin (Cell Signaling, Genscript), β-tubulin and tubulin βIII (Genscript), DIAPH3 (HNH3.1, a generous gift of Dr. Henry Higgs, Dartmouth Medical School).

### Immunofluorescence

Immunostaining was performed as described^5^. Images were acquired on an Axioplan 2 microscope equipped with an Axiocam HR camera (Zeiss), using 63 x or 100 x PlanApo 1.4 NA infinity-corrected objectives.

### Traction force microscopy

DU145 cells (1 × 10^4^) were plated onto collagen I-coated polyacrylamide gels of 1, 11, or 26 kPa stiffnesses, impregnated with 0.2 μm fluorescent yellow beads^39^,^53^. Based on the displacement of fluorescent beads, the elastic properties of the gel substrate, and a manual trace of the contour of the cell, we computed the cell-exerted traction forces using an established method of constrained traction microscopy (TFM)^39^,^53^,^54^,^55^. Root Mean Square (RMS) values of traction in control or DIAPH3-silenced cells were measured at baseline (without nocodazole) or after treatment for 40 min with 2 μM nocodazole. Data were from 3 independent experiments, comprising a total of n = 87, n = 52, and n = 45 control cells and n = 18, n = 27, and n = 21 DIAPH3-silenced cells, at 1, 11, and 26 kPa substrate stiffness. From the traction field, we computed the first-order moment matrix (M). By plotting an ellipse whose semi-axes are determined by the eigenvalues of M and orientation determined by the corresponding eigenvectors of M, we tracked the traction orientation. Finally, we computed the traction polarity based on the ratios of the eigenvalues of M.

### Immunoprecipitation

U87 or DU145 cells, stably expressing GFP or GFP-DIAPH3, were lysed in RIPA buffer (20 mM HEPES, pH 7.4, 1 mM EDTA, pH 8.0, 150 mM NaCl, 1% Igepal, 20 mM sodium fluoride, 20 mM β-glycerol phosphate, 1 mM sodium vanadate, 200 μM PMSF and 60 mM β-octylglucopyranoside). After immunoprecipitation of 500 μg protein, at 4 ^o^C or 25 ^o^C for 3 h with anti-GFP or anti-α-tubulin antibodies and Protein A/G agarose, beads were precipitated, washed, and eluted proteins resolved by SDS-PAGE and immunoblotting. Alternatively, where indicated, U87 cells were pretreated for 24 h with 1 μM of docetaxel or vehicle, and immunoprecipitated as above at 4 ^o^C.

### Identification of DIAPH3 interactomes

U87 cells (1.5 × 10^6^), stably expressing GFP or GFP-DIAPH3, were lysed in RIPA buffer and immunoprecipitated with anti-GFP antibodies at 4 ^o^C, as above. DU145 cells (1.25 × 10^6^), stably expressing GFP or GFP-DIAPH3, were immuno-precipitated at 4 ^o^C or 25 ^o^C. Proteins eluted from the beads were separated in a 10% SDS-PAGE gel, and in-gel reduced, alkylated, and tryptically digested^56^. Tryptic peptides were extracted, concentrated, reconstituted in 0.1% formic acid, separated on a 25 cm EASY-Spray C18 column, and analyzed by an LTQ Orbitrap Elite mass spectrometer (Thermo Scientific). After each survey scan, up to 20 collision-induced dissociation (CID) spectra were acquired in the rapid CID scan mode. For protein identification and quantitation, raw mass spectrometric data were searched against the Uniprot_Human database (released on 02/20/14, including 88647 sequences) with MaxQuant (v 1.3.0.5)^57^ and Andromeda^58^. False discovery rates for protein and peptide identifications were set at 0.01. Identified proteins were quantitated based on their summed ion intensities. All data have been deposited into ProteomeXchange^59^. The DIAPH3 interactome was analyzed using the DAVID bioinformatics database^60^.

### Live cell imaging

DU145 cells were infected with 100 μl of CellLight emGFP-tubulin or 70 μl of TagRFP-tubulin (Life Technologies) in DMEM + 10% FBS lacking phenol red. After 1-2 days at 37 ^o^C, cells were imaged using a 100 x Apo TIRF NA 1.49 oil objective, on a Nikon Ti inverted confocal microscope coupled to a Yokogawa Spinning Disc head.

### Quantitation of MT length

The length of the longest individual MT discernable in a single cell expressing emGFP-tubulin was measured over 10 frames, indicating a 30 s period; 2–3 MT per cell were measured. MT were traced using the freehand line function in ImageJ. The maximum length change was calculated as: [(MT at its longest length – MT at its shortest length) / (MT at its longest length) x 100]. This percentage was determined for 41 MT in control and 35 MT in DIAPH3-silenced DU145 cells.

### Tubulin acetylation induced by MSA

Cells were incubated with varying concentrations of paclitaxel for 30 min at 37 ^o^C, or with 500 nM only of paclitaxel, docetaxel, or epothilone B. Where indicated, after immunoblotting proteins were analyzed by densitometry with ImageJ. In brief, Ac-tubulin band intensity was normalized to that of β-tubulin, in both untreated and MSA-treated conditions. The MSA-induced fold-increase in tubulin acetylation was then calculated, as normalized Ac-tubulin intensity in MSA-treated versus untreated conditions.

### Cytoplasmic and perinuclear tubulin acetylation quantitation

DU145 cells (10^4^) were incubated with 1 μM paclitaxel for 8 h, fixed with 0.1 M cacodylate buffer, pH 6.7, containing 3.7% paraformaldehyde, stained with Ac-tubulin and α-tubulin, and counter-stained with DAPI. Serial optical sections were collected using a TCS SP5 X Supercontinuum confocal microscope (Leica Microsystems) and Plan-Apo 63 × 1.4 oil immersion lens. Images from paclitaxel-treated cells were acquired with a gain of 600 V, and untreated cells with 800 V. Ac-tubulin intensities were normalized using a standard curve of intensities acquired at gains of 600, 700, or 800 V and a second order polynomial model y = a2*x^2 + a1*x + a0, in which y represents the output intensity and x the input intensity. To quantify signal intensity in cytoplasmic and perinuclear shells, global cytoplasmic (Ac-tubulin) and nuclear (Dapi image) masks were defined as described^42^. Perinuclear shells were defined as hollow 3-D spherical volumes adjacent to the nucleus, between the nuclear envelope and a fixed distance ~1/4 of the average nuclear radius away. Perinuclear shells and cytoplasm masks were superimposed onto the tubulin image, and for each cell the two quantities averaged.

### Oregon Green-Paclitaxel 488 (OG-PTX) accumulation

DU145 cells (10^5^) were incubated for 18 h at 37 ^o^C with 1 μM OG-PTX, or DMSO, and lysed in modified RIPA buffer (50 mM Tris-HCl, pH 7.4, 150 mM NaCl, 5 mM EDTA, 1% NP-40, 0.5% sodium deoxycholate, 1% Triton X-100 and 0.2% SDS). Fluorescence was measured on a BMG Labtech FLUOstar Omega platereader, (excitation 490 nm, emission 530 nm), and protein concentration determined using BCA analysis (Pierce).

### MSA-induced cytotoxicity

Cell viability was assessed after treatment with paclitaxel, docetaxel, epothilone B, or DMSO for 4 d by crystal violet. Absorbance was measured at 570 nm.

### Patient cohorts and analysis of DIAPH3 in gene expression profiles

The preprocessed and normalized expression profiles for microarray datasets GSE22226^29^, GSE20271^34^, and GSE25055^35^ were from the Gene Expression Omnibus (GEO) database, or MD Anderson Cancer Center ( http://bioinformatics.mdanderson.org/pubdata.html)^33^. Cohort characteristics are summarized (Supplementary Table S2). Dataset GSE22226 (Agilent G4112A, Trial 1) contained gene expression profiles of a 149-patient cohort, from which 111 patients were selected based on their treatment record of 4 cycles of taxane (paclitaxel or docetaxel) following 4 cycles of doxorubicin/cyclophosphamide (T/AC chemotherapy). Patients treated with Herceptin, or who had not received taxanes after doxorubicin/cyclophosphamide-based therapy, were excluded. Dataset GSE20271 (Affymetrix U133A, Trial 2) contained gene expression profiles of a 178-patient cohort. From this, 91 patients receiving 12 cycles of paclitaxel + 4 cycles of fluorouracil, anthracyclines doxorubicin or epirubicin, and cyclophosphamide (T/FAC chemotherapy), and 87 patients receiving FAC chemotherapy, were selected for analysis. Patients treated with paclitaxel as adjuvant therapy, Xeloda or tamoxifen, or temporal variations in the regimens, were excluded. Dataset GSE25055 (Affymetrix U133A, Trial 3) contained gene expression profiles of a 310-patient cohort, consisting of 227 cases treated with 12 cycles of paclitaxel + 4 cycles of fluorouracil, doxorubicin and cyclophosphamide and 83-cases treated either with 4 cycles of doxorubicin and cyclophosphamide + 4 cycles of paclitaxel (n = 60) or docetaxel (n = 18), or for whom the taxane was not specified (n = 5). Clinical information was extracted from the GEO database.

Additionally, the relationship of DIAPH3 expression to overall survival (OS) was assessed using the Oncomine® Power Tools Clinical Outcomes Beta v3.1 Library (Life Technologies). Microarray profiling data for the two selected studies^36^,^37^ were exported and segmented into four expression quartiles based on the transcript levels of the DIAPH3 locus. Data were then subdivided into categories of “low” (<25^th^ percentile) or “high” (≥25^th^ percentile) DIAPH3 expression, and correlated with clinical outcome as survival curves obtained by Kaplan-Meier analysis. A Log Rank test was used for statistical comparison.

### Statistical analyses

Normalized DIAPH3 intensities were used to assess the association of DIAPH3 gene expression with OS and/or RFS. Cox proportional hazards regression was utilized when correlating DIAPH3 expression with OS and RFS. Wilcoxon Rank-Sum tests were applied when examining differences in DIAPH3 expression for patients achieving clinically-verified or DLDA30-predicted pCRs, versus those who did/were not. Fisher’s exact test was used to evaluate the number of patients predicted to achieve pCR by the DLDA-30 signature. MT length measurements were evaluated with a Mann-Whitney test. For remaining biological and biochemical studies, a two-sided Student’s t-test was used.

## Additional Information

**How to cite this article**: Morley, S. *et al.* Regulation of microtubule dynamics by DIAPH3 influences amoeboid tumor cell mechanics and sensitivity to taxanes. *Sci. Rep.* **5**, 12136; doi: 10.1038/srep12136 (2015).

## Supplementary Material

Supplementary Information

Supplementary Information

Supplementary Information

Supplementary Information

Supplementary Information
