# Supplementary material for: Regulation of microtubule dynamics by DIAPH3 influences amoeboid tumor cell mechanics and sensitivity to taxanes
Source: Sci Rep. 2015 Jul 16;5:12136. doi: 10.1038/srep12136 (PMC4503992; doi:10.1038/srep12136)
Supplement: Supplementary Information [file srep12136-s1.pdf]

Regulation of microtubule dynamics by DIAPH3 influences amoeboid tumor cell mechanics and sensitivity to taxanes

Samantha Morley, Sungyong You, Sara Pollan, Jiyoung Choi, Bo Zhou, Martin H Hager, Kenneth Steadman, Cristiana Spinelli, Kavitha Rajendran, Arkadiusz Gertych, Jayoung Kim, Rosalyn M Adam, Wei Yang, Ramaswamy Krishnan, Beatrice S Knudsen, Dolores Di Vizio, and Michael R Freeman

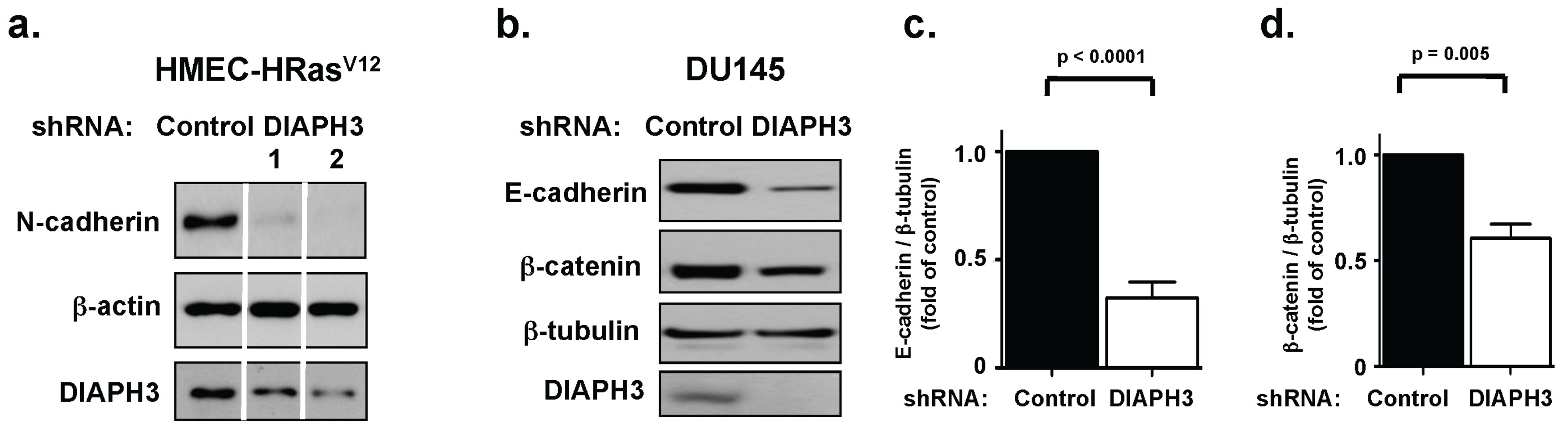
a.
HMEC-HRas^{V12}
shRNA: Control DIAPH3
1 2
N-cadherin
β-actin
DIAPH3
b.
DU145
shRNA: Control DIAPH3
E-cadherin
β-catenin
β-tubulin
DIAPH3
c.
p < 0.0001
E-cadherin / β-tubulin (fold of control)
1.0
0.5
0
shRNA: Control DIAPH3
d.
p = 0.005
β-catenin / β-tubulin (fold of control)
1.0
0.5
0
shRNA: Control DIAPH3

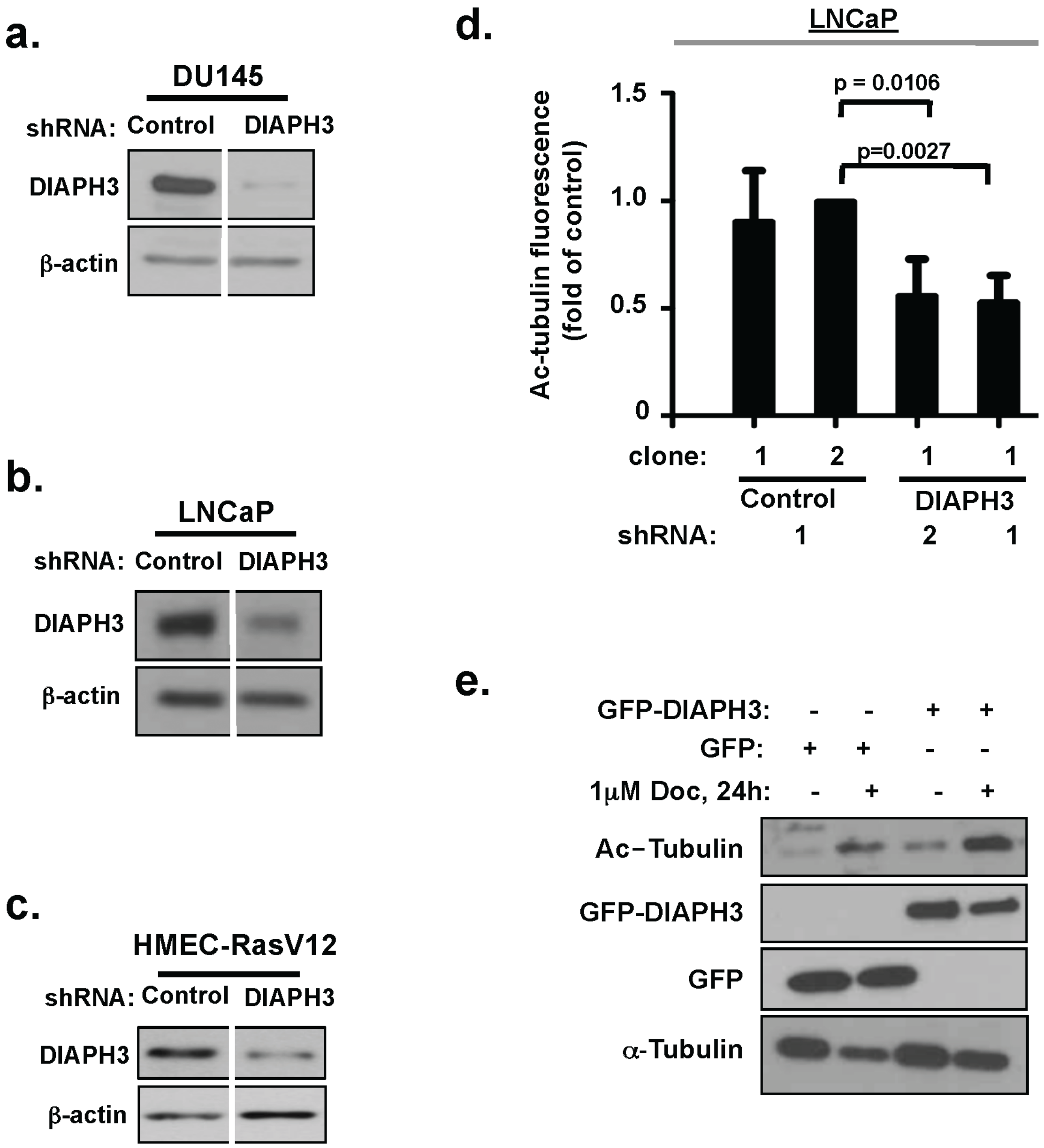
a.
DU145
shRNA: Control DIAPH3
DIAPH3
β-actin
b.
LNCaP
shRNA: Control DIAPH3
DIAPH3
β-actin
c.
HMEC-RasV12
shRNA: Control DIAPH3
DIAPH3
β-actin
d.
LNCaP
Ac-tubulin fluorescence (fold of control)
1.5
1.0
0.5
0
p = 0.0106
p=0.0027
clone: 1 2 1 1
Control
DIAPH3
shRNA: 1 2 1
e.
GFP-DIAPH3: - - + +
GFP: + + - -
1μM Doc, 24h: - + - +
Ac−Tubulin
GFP-DIAPH3
GFP
α-Tubulin

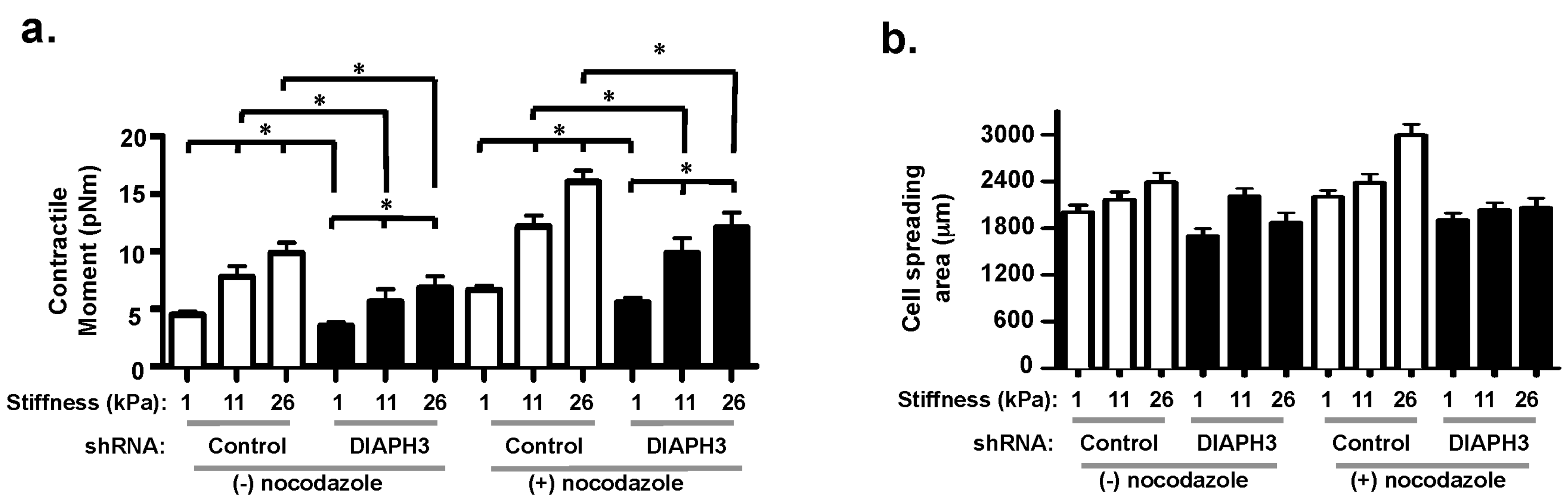

a.
*
*
*
*
*
*
*
*
20
15
10
5
0
Contractile Moment (pNm)
Stiffness (kPa): 1 11 26 1 11 26 1 11 26 1 11 26
shRNA: Control DIAPH3 Control DIAPH3
(-) nocodazole
(+) nocodazole
b.
3000
2400
1800
1200
600
0
Cell spreading area (µm)
Stiffness (kPa): 1 11 26 1 11 26 1 11 26 1 11 26
shRNA: Control DIAPH3 Control DIAPH3
(-) nocodazole
(+) nocodazole

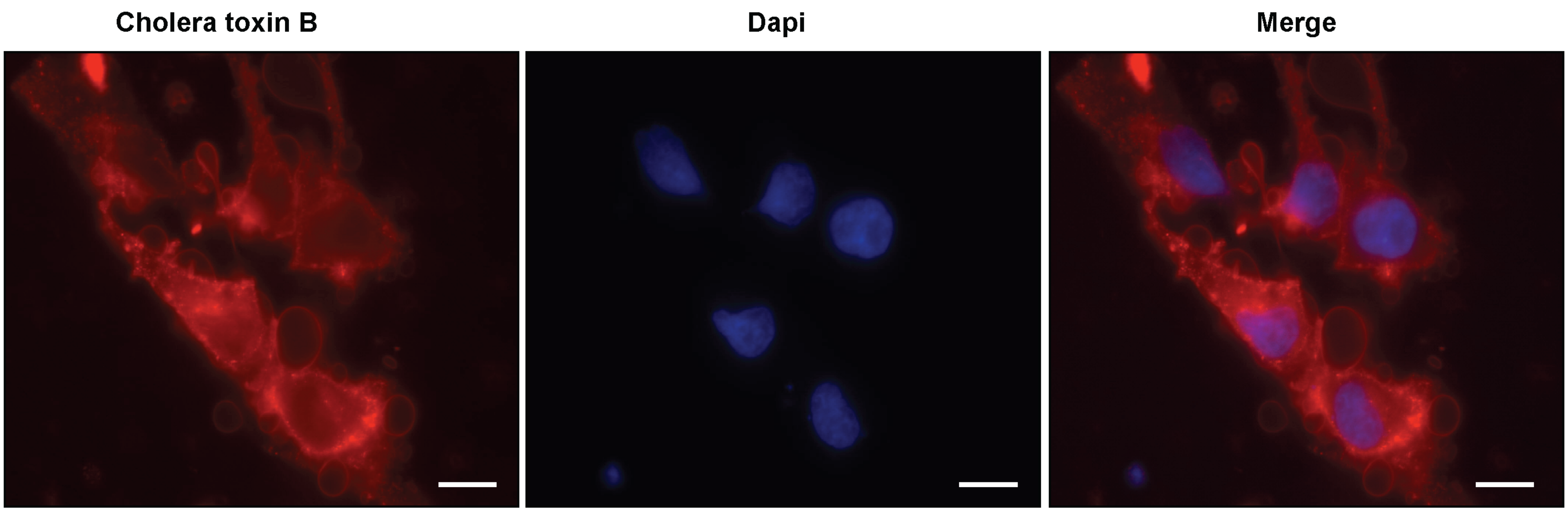
Cholera toxin B
Dapi
Merge

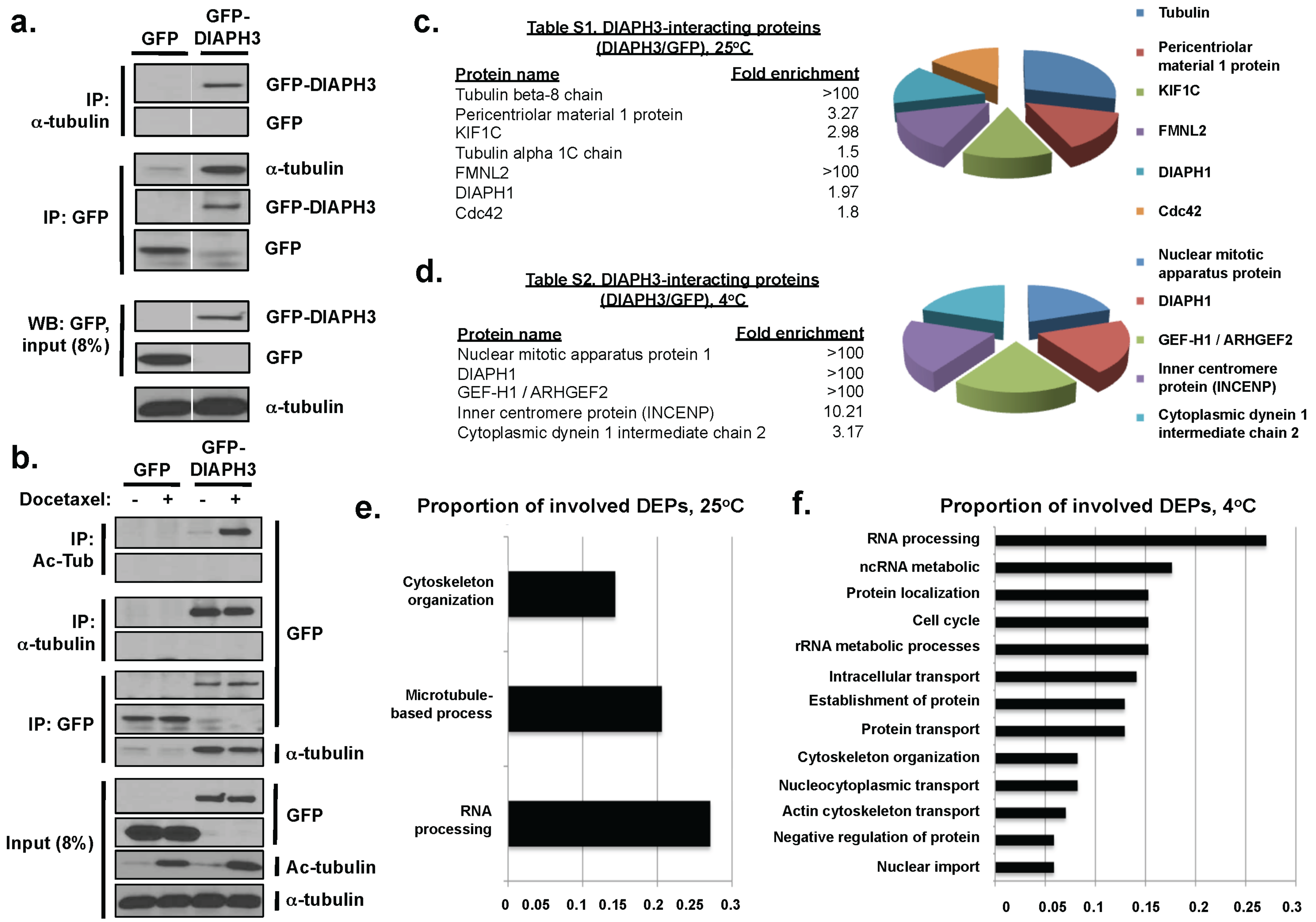

a.
GFP
GFP-DIAPH3
IP: α-tubulin
GFP-DIAPH3
GFP
IP: GFP
α-tubulin
GFP-DIAPH3
GFP
WB: GFP, input (8%)
GFP-DIAPH3
GFP
α-tubulin
b.
GFP
GFP-DIAPH3
Docetaxel: - + - +
IP: Ac-Tub
IP: α-tubulin
IP: GFP
Input (8%)
GFP
α-tubulin
GFP
Ac-tubulin
α-tubulin
c.
Table S1. DIAPH3-interacting proteins (DIAPH3/GFP), 25°C
Protein name | Fold enrichment
Tubulin beta-8 chain | >100
Pericentriolar material 1 protein | 3.27
KIF1C | 2.98
Tubulin alpha 1C chain | 1.5
FMNL2 | >100
DIAPH1 | 1.97
Cdc42 | 1.8
Tubulin
Pericentriolar material 1 protein
KIF1C
FMNL2
DIAPH1
Cdc42
d.
Table S2. DIAPH3-interacting proteins (DIAPH3/GFP), 4°C
Protein name | Fold enrichment
Nuclear mitotic apparatus protein 1 | >100
DIAPH1 | >100
GEF-H1 / ARHGEF2 | >100
Inner centromere protein (INCENP) | 10.21
Cytoplasmic dynein 1 intermediate chain 2 | 3.17
Nuclear mitotic apparatus protein
DIAPH1
GEF-H1 / ARHGEF2
Inner centromere protein (INCENP)
Cytoplasmic dynein 1 intermediate chain 2
e.
Proportion of involved DEPs, 25°C
Cytoskeleton organization
Microtubule-based process
RNA processing
0 0.05 0.1 0.15 0.2 0.25 0.3
f.
Proportion of involved DEPs, 4°C
RNA processing
ncRNA metabolic
Protein localization
Cell cycle
rRNA metabolic processes
Intracellular transport
Establishment of protein
Protein transport
Cytoskeleton organization
Nucleocytoplasmic transport
Actin cytoskeleton transport
Negative regulation of protein
Nuclear import
0 0.05 0.1 0.15 0.2 0.25 0.3

Supplementary Table S1. Correlation of Diaphanous-related formin expression with GI_{50} in response to MT-stabilizing and depolymerizing agents*.

NSC	*Drug*	DIAPH1 (*Pearson Correlation Coefficient)*	DIAPH2 (*Pearson Correlation Coefficient)*	DIAPH3 (*Pearson Correlation Coefficient)*
	(MT Stabilizing Agent)			
125973	Paclitaxel	0.163	0.037	-0.241
600222	Benzenepropanoic acid	0.258	-0.028	-0.205
608832	Taxol derivative	0.102	0.099	-0.217
628503	Docetaxel	0.192	0.033	-0.088
656178	7-Epi-10-deacetylbaccatin II	0.215	0.12	-0.3
658831	Taxol derivative	0.122	-0.017	-0.259
661746	2-Debenzoyl-2-(3’,5’-difluorobenzoyl)-15(16)-anhydro-11(15->1)-abetaxol	0.195	0.06	-0.16
664402	Taxol derivative	0.161	0.141	-0.271
664404	Taxol derivative	0.075	-0.074	-0.348
666608	Taxol derivative	0.324	0.112	-0.15
671867	Taxol derivative	0.258	0.08	-0.176
671870	Taxol derivative	0.366	0.118	-0.173
673187	Taxol derivative	0.257	0.039	-0.27
673188	Taxol derivative	0.253	-0.051	-0.2
747973	Ixabepilone	0.283	0.089	-0.088
	(MT depolymerizing agent)			
757	Colchicine	0.043	0.106	-0.144
33410	Colchicine derivative	0.013	0.208	-0.065
49842	Vinblastine sulfate	0.058	0.152	-0.279
67574	Vinblastine sulfate	-0.006	0.041	-0.156
89201	Estramustine phosphate sodium	0.258	0.09	-0.118
332598	Rhizoxin	-0.146	0.053	-0.141
361792	Thiocolchicine	0.004	0.263	-0.074
376128	Dolastatin 10	0.251	0.1	-0.332
406042	Allocolchicine	-0.06	0.083	-0.014
608210	Vinorelbine tartrate	0.187	-0.104	-0.139
609395	Halichondrin B	-0.079	0.079	-0.063
707389	Eribulin mesylate	0.321	-0.003	-0.243

* Tubulin targeted agents were selected from the NSC drug screen database and segregated into stabilizing or depolymerizing (italics) agents. Cross-correlation analysis was then performed using the CellMiner ™ database tool against DIAPH1, DIAPH2 and DIAPH3 Z-score transcript data within the NCI-60 database. The analysis indicates that lower DIAPH3 expression correlates to decreased resistance to both stabilizing and depolymerizing tubulin agents, while lower DIAPH1 expression correlates with increased resistance. DIAPH2 results were inconclusive.

Supplementary Table S2. Characteristics of clinical cohorts utilized.

		1	2	3	4
	**BCa cohort**	Esserman et al.	Tabchy et al.	Hatzis et al.	Hess et al.
	**GEO Accession**	GSE22226	GSE20271	GSE25055	N/A http://bioinformatics.mdanderson.org/pubdata.html
	**Array platform**	Agilent G4112A	Affymetrix U133A	Affymetrix U133A	Affymetrix U133A
Characteristics of cohort	**Total pts**	111	91	310	182
	**Age**	Median = 47	Mean = 51.5	Mean = 50	Median = 52
	**Average grade**	3	2/3	3	3
	**Average stage**	T2B/T3A	T2	T2	T2
	**% TNBC**	30%	N/A	N/A	N/A
Treatment	**Tx group 1 (# patients)**	4 x AC + 4x taxane (111)	12 x weekly paclitaxel + 4 x 3-weekly FAC/FEC (91)	12 x weekly paclitaxel + 4 x FAC (227)	12 x weekly paclitaxel + 4 x FAC (153)
	**Tx group 2 (# patients)**			4 x 3-weekly docetaxel + 4 x AC (83)	4 x 3-weekly docetaxel + 4 x FAC (29)
	**Neo-adjuvant**	X	X	X	X
Outcomes data	**pCR**	X	X	X	X
	**DLDA-30 Signature**		DLDA30 score (predictive score derived from 27-gene, 31-probe signature)		DLDA30 score (predictive score derived from 27-gene, 31-probe signature)
	**PFS**	X		X	
	**OS**	X			

Supplementary Figure Legends.

<u>Supplementary Figure S1. DIAPH3 loss suppresses features of the EMT.</u> Levels of N-cadherin (A), E-cadherin (B), or β-catenin (B) were assessed by immunoblotting from lysates from HRAS-transformed HMEC cells (A) or DU145 PCa (B-D) cells. (C-D) Immunoblots were analyzed by densitometry using ImageJ. E-cadherin or β-catenin intensities were normalized to β-tubulin. Normalized intensities from 3 independent trials were then averaged and plotted as the fold-change in intensity relative to non-silenced cells. Shown are means ± standard deviation.

<u>Supplementary Figure S2. DIAPH3 expression modulates tubulin acetylation.</u> A-C, Silencing of DIAPH3 in DU145 **(A)**, LNCaP **(B)**, or HRasV12-transformed HMEC **(C)** cells. **D.** The intensity of Ac-tubulin fluorescence in LNCaP cells was quantified using ImageJ analysis software. Data shown are normalized to tubulin acetylation in control cells, clone 2. **E.** U87 cells stably expressing GFP or GFP-DIAPH3 were incubated with 1 μM docetaxel for 24h, and Ac-tubulin levels assessed by immunoblotting.

<u>Supplementary Figure S3. Contractile moment and cell spreading area of non-silenced and DIAPH3-silenced DU145 cells.</u> A. Contractile moment, an integrated measure of traction force and cell spreading area, was determined in the absence or presence of 2 μM nocodazole, as described in the Methods (and [55]). **B.** Cell spreading area was monitored prior to or following incubation with 2 μM nocodazole.

<u>Supplementary Figure S4. Amoeboid properties of U87 glioblastoma cells.</u> U87 cells were stained with Cholera toxin B and counterstained with Dapi. Images are

representative of at least 10 fields, from 3 independent experiments. Note blebbing, amoeboid morphology of U87 cells. Scale bars, 10 μm.

<u>Supplementary Figure S5. Interaction of DIAPH3 with proteins relevant to the MT cytoskeleton.</u> A. Association of DIAPH3 with α-tubulin in U87 cells. Reciprocal co-immunoprecipitations of GFP or GFP-DIAPH3 with α-tubulin. **B.** Association of DIAPH3 with Ac-tubulin in U87 cells. Cells expressing GFP or GFP-DIAPH3 were incubated with 1 μM Docetaxel or vehicle for 24h prior to lysis and reciprocal co-immunoprecipitation with GFP or Ac-tubulin. **C.** Proteins preferentially interacting with GFP-DIAPH3 over GFP at 25°C (MT intact). *Right,* pie chart illustrating the fraction of each subtype of MT-relevant proteins to the total number of MT-relevant proteins detected at 25°C. **D.** Proteins preferentially interacting with GFP-DIAPH3 over GFP at 4°C (MT depolymerized). *Right,* pie chart illustrating the fraction of each subtype of MT-relevant proteins to the total number of MT-relevant proteins detected at 4°C. **E.** Gene Ontology analyses of differentially-expressed proteins (DEPs) from (C). **F.** Gene Ontology analyses of DEPs from (D). Note enrichment of DIAPH3 interaction with cytoskeleton organization-associated proteins at 25°C vs. 4°C. Also note emergence of DIAPH3 interaction with MT-based process-associated proteins at 25°C, at which MT are polymerized.

Supplementary Movie Legends.

<u>Supplementary Movies 1, 2. Control shRNA, emGFP-tubulin.</u> DU145 control cells were infected with CellLight emGFP-tubulin and imaged by spinning disc confocal microscopy. Individual frames were acquired at 3 s intervals with a Hamamatsu Orca ER

digital CCD. Movies are comprised of 41 frames each, of duration 123 s, in Mpeg-4 format at 7 frames per second, using ImageJ freeware.

Supplementary Movies 3, 4. DIAPH3 shRNA, emGFP-tubulin. DU145 cells silenced for DIAPH3 were infected with CellLight emGFP-tubulin and imaged by spinning disc confocal microscopy. Individual frames were acquired at 3 s intervals with a Hamamatsu Orca ER digital CCD. Movies are comprised of 41 frames each, of duration 123 s, in Mpeg-4 format at 7 frames per second, using ImageJ freeware.
